# Large animal model species in pluripotent stem cell therapy research and development for retinal diseases: a systematic review

**DOI:** 10.3389/fopht.2024.1377098

**Published:** 2024-08-26

**Authors:** Julia-Sophia Bellingrath, Kang V. Li, Kanza Aziz, Jessica M. Izzi, Ying V. Liu, Mandeep S. Singh

**Affiliations:** ^1^ Nuffield Laboratory of Ophthalmology, Nuffield Department of Clinical Neurosciences, University of Oxford, Oxford, United Kingdom; ^2^ Wilmer Eye Institute, Johns Hopkins University School of Medicine, Baltimore, MD, United States; ^3^ Department of Ophthalmology, Massachusetts Eye and Ear, Harvard Medical School, Boston, MA, United States; ^4^ Department of Molecular and Comparative Pathobiology, Johns Hopkins University School of Medicine, Baltimore, MD, United States; ^5^ Department of Genetic Medicine, Johns Hopkins University, Baltimore, MD, United States

**Keywords:** retinal degeneration, surgery techniques, stem cell transplantation, preclinical (*in vivo*) studies, immunosuppression

## Abstract

**Aim:**

Retinal cell therapy modalities, in the category of advanced therapy medicinal products (ATMPs), are being developed to target several retinal diseases. Testing in large animal models (LAMs) is a crucial step in translating retinal ATMPs into clinical practice. However, challenges including budgetary and infrastructure constraints can hinder LAM research design and execution. Here, to facilitate the comparison of the various LAMs in pluripotent retinal cell therapy research, we aimed to systematically evaluate the species distribution, reported scientific utility, and methodology of a range of LAMs.

**Methods:**

A systematic search using the words retina, stem cell, transplantation, large animal, pig, rabbit, dog, and nonhuman primate was conducted in the PubMed, Embase, Science Direct and GoogleScholar databases in February 2023.

**Results:**

We included 22 studies involving pluripotent stem cells (induced pluripotent stem cells or human embryonic stem cells) in LAMs, including non-human primates (NHP), pigs, dogs, and rabbits. Nearly half of the studies utilized wild-type animal models. In other studies, retinal degeneration features were simulated via laser, chemical, or genetic insult. Transplants were delivered subretinally, either as cell suspensions or pre-formed monolayers (with or without biodegradable scaffolding). The transplanted cells dose per eye varied widely (40,000 – 4,000,000 per dose). Cells were delivered via vitrectomy surgery in 15 studies and by an “ab externo” approach in one study. Structural outcomes were assessed using confocal scanning laser ophthalmoscopy imaging. Functional outcomes included multifocal electroretinogram and, in one case, a measure of visual acuity. Generally, cell suspension transplants exhibited low intraretinal incorporation, while monolayer transplants incorporated more efficiently. Immune responses posed challenges for allogeneic transplants, suggesting that autologous iPSC-derived transplants may be required to decrease the likelihood of rejection.

**Conclusion:**

The use of appropriate LAMs helps to advance the development of retinal ATMPs. The anatomical similarity of LAM and human eyes allows the implementation of clinically-relevant surgical techniques. While the FDA Modernization Act 2.0 has provided a framework to consider alternative methods including tissue-on-a-chip and human cell culture models for pharmacologic studies, LAM testing remains useful for cell and tissue replacement studies to inform the development of clinical trial protocols.

## 1 Introduction

Cell and tissue regeneration approaches to treat blinding diseases are in clinical development, most recently with the launch of an important clinical trial at the National Institutes of Health. In this trial, patients with geographic atrophy secondary to dry age-related macular degeneration (AMD), will each receive the implantation of a patch of retinal tissue generated using autologous cells (source: www.nei.nih.gov, accessed 24 November 2023). Several previous clinical trials have also investigated stem cell-based regenerative therapies for retinal degeneration (1–5). Efforts such as these could move the needle significantly for human health, as blinding diseases such as geographic atrophy are bereft of regenerative treatment options presently.

The groundwork for the use of pluripotent stem cells in regenerative medicine clinical trials was laid in 1998 when human Embryonic Stem Cells (hESC) were isolated from the inner cell mass (ICM) of the blastocyst of a human embryo (6). Barely 10 years later, when it was shown that adult somatic cells could be reprogrammed to pluripotency, induced pluripotent stem cells (iPSC) emerged as a less controversial and widely available alternative (7, 8). Contrary to hESCs, which can serve only as an allogeneic stem cell source, iPSC can potentially be used as an autologous, non-immunogenic, patient-specific stem cell source. Additionally, they enable the creation of *in vitro* disease models to better understand pathogenesis of degenerative diseases (9). The most enticing potential application of pluripotent stem cells in vision science is as a substrate for therapeutic transplantation to treat degenerative retinal diseases.

Development of regenerative approaches for the human retina are paramount, since, contrary to non-mammalian vertebrates, higher vertebrates cannot efficiently regenerate neurons after degenerative or traumatic damage to the retina (10). In many ways, the eye, and more specifically, the retina, is an ideal target organ for stem cell therapy, which is reflected in retinal stem cell therapy being at the forefront of clinical stem cell trials (11). It is an easily accessible target by conventional surgical techniques. The transplanted cells can be monitored by non-invasive imaging methods such as optical coherence tomography (OCT) and scanning laser ophthalmoscopy (SLO). In addition, subjective and objective endpoints such as visual acuity, perimetry and electroretinography (ERG) are available to track functional outcomes. The contralateral eye, in some instances, can be regarded as a control. Although reports of exist of intraocular cell migration (12), there is no evidence of stem cell progeny migrating outside of the eye, making activation of a systemic immune response relatively unlikely, thus limiting the risk for systemic adverse effects. In comparison to degenerative central nervous system diseases, a small number of transplanted cells is required to improve to restore visual function in eye diseases (13).

Most importantly, due to the current lack of curative therapies for many of the most common blinding degenerations, including age-related macular degeneration (AMD), Stargardt disease, Best disease and retinitis pigmentosa (RP), there is a huge unmet need for retinal regenerative treatments. While gene therapy is emerging as a viable treatment for a several IRDs (14, 15), stem cell therapy has the potential advantage of offering a more broadly-applicable, mutation-independent treatment for a wide variety of degenerative retinal diseases.

Human retinal stem cell trials are underway, and in general they have demonstrated favorable safety profiles (1, 2, 16, 17). Data from mouse models have been widely used to demonstrate proof of concept (18–20). Rodent models have the advantage of reproducing quickly with short generation intervals and typically exhibiting high fertility. Furthermore, they can be maintained cost-efficiently and can be genetically manipulated with relative ease. But the evolutionary distance of the mouse eye to its human counterpart is reflected in the mouse eye’s much smaller anatomical scale, rod-dominated vision and the absence of a macula or a visual streak (21). In addition, mouse models often fail to fully reproduce human disease phenotypes (22, 23).

To bridge the translational gap between preclinical rodent trials and clinical application, large animal models (LAMs), are emerging to more comprehensively evaluate the translational potential of stem cell therapies. While a formal definition of LAM is lacking, mammalian animal models that do not fall under the category of rodents are commonly referred to as LAM (22, 23). Commonly used LAM in stem cell therapy research include rabbits, dogs, pigs and nonhuman primates (NHP) (24–27), which are covered in this review. The relevance of the cat model is discussed only briefly owing to its generally limited use in this field.

Husbandry for LAMs is more expensive and labor intensive, requires longer breeding times and fewer offspring than rodents, and LAM disease models are scarce. However, LAMs offer several crucial advantages: their eyes are more closely approximate the human eye in terms of anatomical dimensions, physiology and immune response characteristics (23). These aspects uniquely position them as enabling tools to evaluate methodology and outcomes of human stem cell transplantation approaches. The anatomical similarity allows for the almost direct translation of the preclinical surgical protocol to a clinical trial, as well as a better estimation of the cell dose that is required to achieve a functional effect. Furthermore, the presence of a macula or fovea in certain LAMs enables more accurate modeling of therapy directed at the macula (including for AMD and Stargardt disease which are among the prime targets of stem cell therapy development). Due to their longer life span than rodents, LAMs can also facilitate longitudinal follow-up to evaluate relatively long-term outcomes.

The principal aims of this systematic review are to assess the species distribution, scientific utility, and methodology of LAMs in retinal cell therapy research. Here, we will also provide an overview of the main findings of preclinical retinal stem cell studies that have been performed in LAMs. In addition, we will evaluate their structural characteristics relative to the human and rodent eyes. This review will thus provide a comprehensive characterization of the range of available LAMs for the eye and an elucidation of the similarities and differences between them.

## 2 Materials and methods

A systematic search for large animal models and retinal stem cell therapy using the words retina, stem cell, transplantation, large animal, pig, rabbit, dog, and nonhuman primate was conducted in the PubMed, Embase, Science Direct and GoogleScholar databases in February 2023. Twenty-two published studies matching these criteria were published from 2011 to 2022. One study describing the generation of an albino rabbit model of geographic atrophy (GA) was excluded due to it being purely a description of the methodology (28), but the preclinical study utilizing this rabbit GA model was included (29). Only those studies that used pluripotent stem cells were included in this review, while those using multipotent cells such as mesenchymal bone marrow cells, forebrain progenitor cells, or lineage-committed retinal progenitor/stem cells were excluded (30–33). Rodent studies were included for comparison purposes only.

## 3 Results

### 3.1 Overview of properties of the human, rodent and LAM eyes

**Figure 1:**
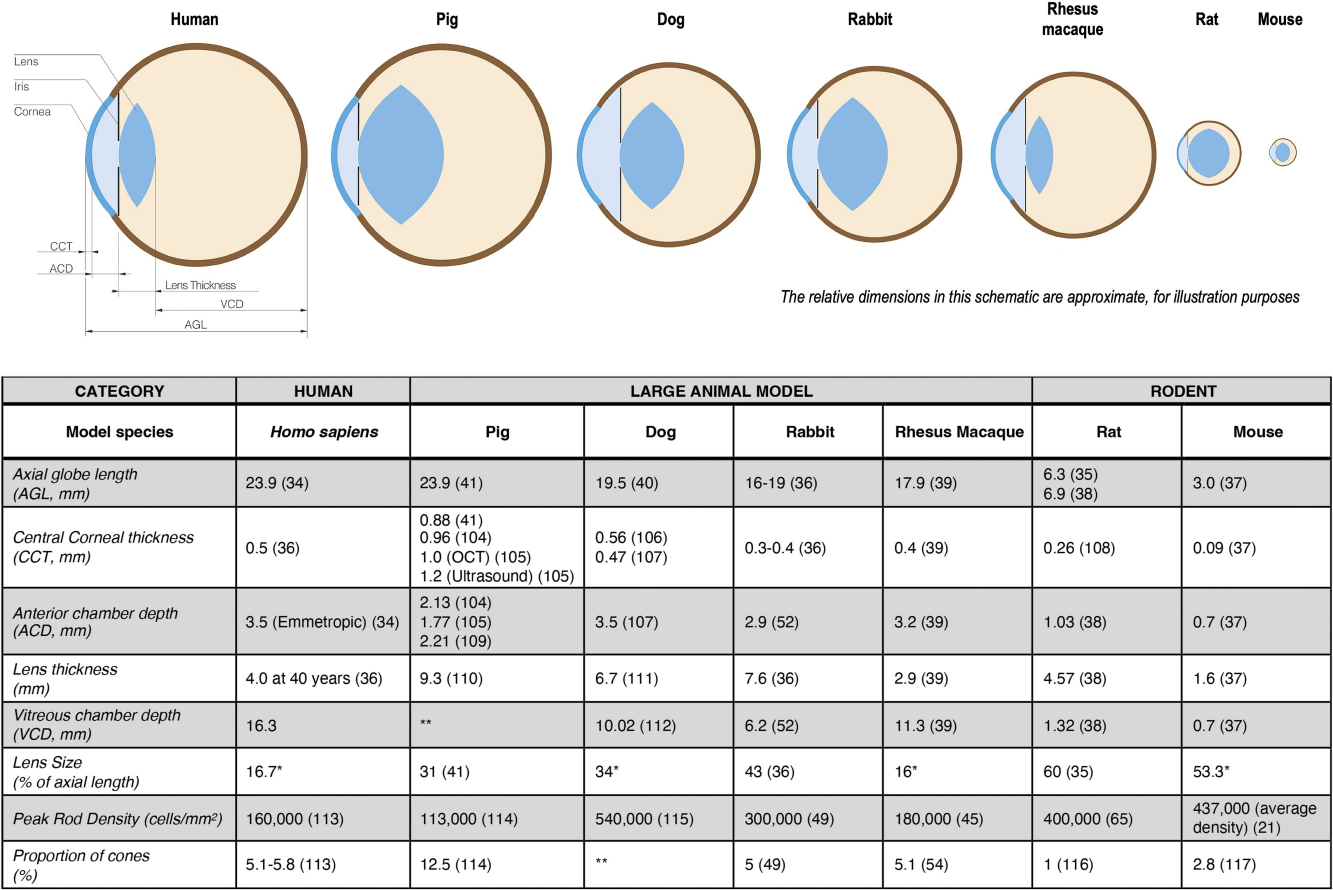
Ocular morphology characteristics of large animal model (lam) eyes compared with those of humans and rodents. When differing values were found in the literature, they are presented denoting the respective references. *Lens size (percentage of axial length) was calculated using the given values for axial length and the lens thickness; **Data not found in the literature review (35–41, 45, 49, 52, 54, 65, 104–117).

When comparing the human eye to that of rodents and LAMs (**Figure 1**), two main attributes can be considered: (1) the size and proportions of the eye and its compartments, and (2) the distribution and ratio of rod and cone photoreceptors within the eye.

Perhaps the most obvious difference between the rodent and human eye is size. The axial length of the human eye (average, 23.9 mm (34)) is eight times longer than that of the mouse, and over three times longer than that of the rat (34–38). The lens of the rodent eye occupies more than half of its eye volume (53 and 60% of the mouse and rat eyeball, respectively (35, 37)) and therefore, the vitreous chamber of a rodent is much smaller and narrower than that of a human.

Of the four LAMs considered here that have all been used for translational pre-clinical studies of pluripotent stem cell research in the retina (see **Figure 1**), the pig eye (23.9 mm) – not the eye of the dog (19.5 mm) nor NHP (17.9 mm) – is closest in size to the human eye, giving it a possible advantage as a model for studies primarily addressing the technical aspects of surgical delivery of the cells (39–41).

Compared to the overall physical size of a rabbit, its eye is large and its axial length (16 – 19 mm) is about 70% of a human eye (36). Rabbits are sometimes considered a less costly surgical model than pig, but one factor that may hinder the use of a rabbit eye as a surgical model is its relative compression in the anteroposterior diameter, whereas the human eye is more spherical (36). For experimental purposes, it is important to note that a rabbit has a retrobulbar venous plexus, thus requiring a transconjunctival rather than a transpalpebral approach to enucleation (42).

Not only do rodent eyes differ considerably in size compared to humans, but they also have a higher rod density than humans. While the rod to cone ratio in humans is 20:1, it is 35:1 in the rodent model (21, 43) This is reflective of the nocturnal circadian rhythm of rats and mice, whereas humans are diurnal mammals. More importantly, mice and rats lack a cone rich retinal region such as the primate macula or visual streak in pigs and rabbits (44). This poses a particular challenge when studying translational aspects of conditions that mainly affect the macula, such as AMD and macular dystrophies.

**Figure 2:**
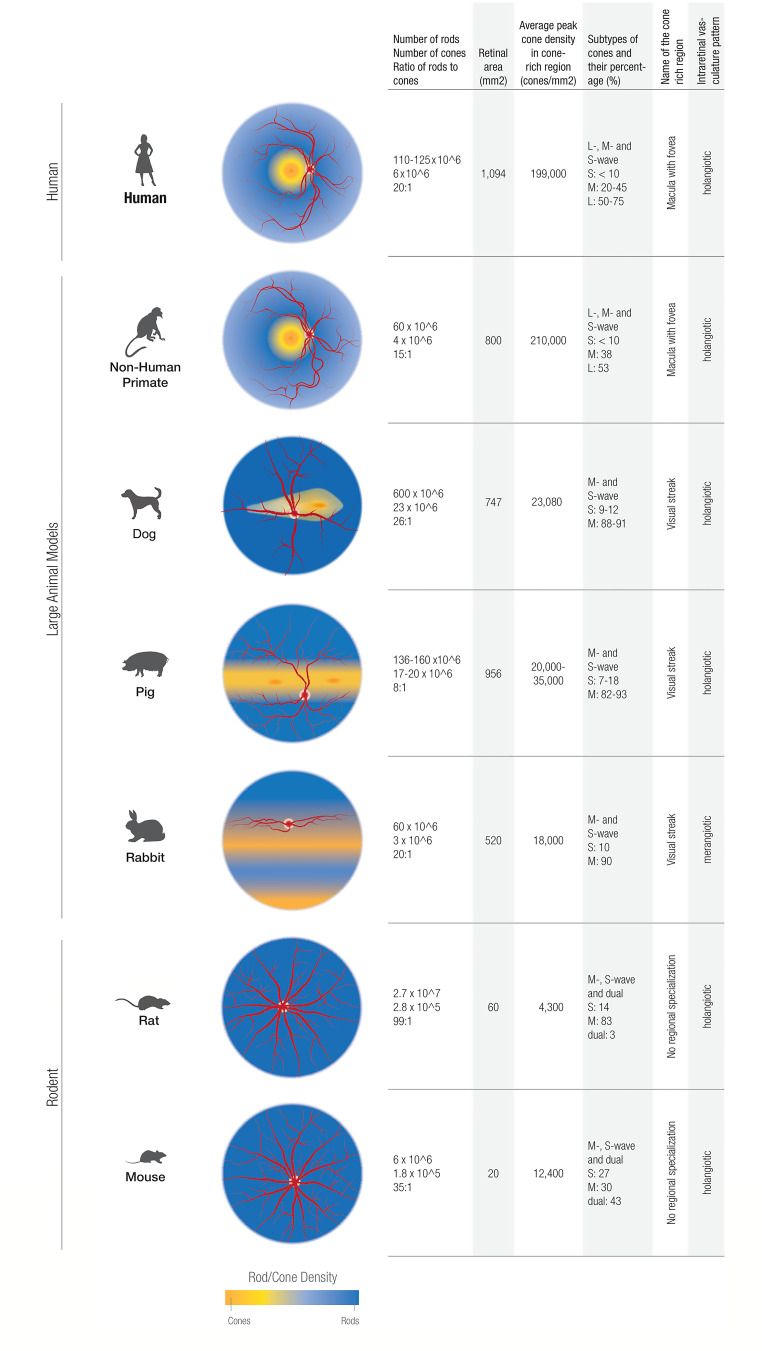
Comparing cone characteristics in the retinas of humans, rodents, and large animal models studied in this review. The nonhuman primate models generally provide a more faithful representation of the central-to-peripheral gradient of cone/rod density as found in humans. In contrast, the dog, pig, and rabbit models show cone/rod density gradients generally oriented along the horizontal meridia.

The NHP mimics the human eye most closely in regard to photoreceptor distribution. As a cone-dense region, both NHP and humans have a fovea, which contains an all-cone foveola (45). Even though the average peak cone density in this cone-rich region displays a high variability between individuals it is similar between NHP and humans (**Figure 2**): humans have 199,000 (100,000 – 324,000) cones/mm^2^ and NHP 210,000 (190,000 – 260,000) cones/mm^2^ (43, 46). Pigs, dogs and rabbits do not have a macula, but instead have a cone-rich visual streak (44, 47, 48). Cones in the visual streak of pigs and rabbits are much less dense than in the fovea of humans and NHP. The pig displays an average density of 20,000-35,000 cones/mm^2^, and has two locations within its visual streak where the cone density is increased up to 40,000 cones/mm^2^ (47). In its visual streak, the rabbit exhibits a peak density of 18,000 cones/mm^2^ and dog has about 23,000 cones/mm^2^ (48, 49).

When comparing rod to cone ratios, NHP and rabbits most closely mimic the rod to cone ratio in humans. In NHP, the rod to cone ratio is 15:1 (50). The rabbit exhibits an identical rod to cone ratio to the human one: 20:1 (43, 51, 52). The dog retina is rod dominated with a rod-to-cone ratio of 41:1 in the inferior peripheral retina and 23:1 in the area centralis (48). Pigs, on the other hand, have a much higher proportion of cones and therefore a rod to cone ratio of 8:1 (47, 53). NHP show a percentage of cones in the macula (5.1%) that is approximately equal to humans (54). In addition to the visual streak, the rabbit exhibits another crescent-shaped, cone dense region in the inferior retina, which is populated exclusively by S-cones and is thus termed “blue streak” (55). Cone density of the blue streak is of the same order of magnitude as it is in the visual streak (55).

As well as sharing a cone distribution pattern, NHP exhibit the same cone subtypes as humans. Both humans and NHP are generally trichromats, with an added long wave (L) cone pigment, which is sensitive to red light (56). In contrast, non-primate mammals are most often dichromats and exhibit just two opsins sensitive to middle (M) and short (S) wavelengths. In the mouse, dual cones, which co-express both S- and M- opsins, outnumber both genuine S- and M- opsin cones (57).

In humans, the number of cone subtypes vary greatly between individuals (58, 59), but generally, S-opsin cones constitute less than 10% of the cones in the primate retina (58, 60). L – opsins constitute between 50 – 75% cones, whereas M-opsins make up about 20-45% of cones in humans (58). Generally, S-cones make up less than 10% of the cone population in mammals, with L-cones being the predominant cone type. A ratio of S to M cones of 1:10 can be used as a rule of thumb in most mammals (61), but some notable exceptions of cone proportions within the retina of animal models should be mentioned: the rabbit retina contains a “blue streak”, a crescent shaped, S- cone-dense area in the inferior retina (55) and in the mouse, the inferior retina contains exclusively S-cones (62). Regarding the cone subtype distribution in the foveola vicinity, in Rhesus macaque retinas, there is a predominance of M/L cones. Conversely, in the periphery, there is a greater proportion and density of S cones, akin to the distribution pattern in human retinas (45, 63).

The presence of intraretinal vasculature is a defining feature of mammals (64). Of the animals discussed in this paper, the rabbit is unique in its merangiotic intraretinal vasculature pattern in which the intraretinal blood vessels are confined to a linear horizontal streak on both sides of the optic disc and are accompanied by myelinated nerve fibers (42). Consequently, in the rabbit, and Leporidae in general, the inner retina is supplied to a large part by the choriocapillaris (42). In species with a holangiotic intraretinal vasculature pattern, such as carnivores, primates and most rodents, the blood vessels are distributed throughout most of the neurosensory retina, resulting in the inner retina being supplied by central retinal and cilioretinal arteries and the outer retina by the choriocapillaris (65).

A major drawback of the use of LAMs, which is reflected in the preclinical studies reviewed here, is the limited range of models of retinal degeneration that are available for the majority of LAM (with the notable exception of the dog (66)). This is particularly relevant since some data appear to suggest that stem cells appear to integrate more efficiently in healthy, wild-type model eyes than in disease models (24).

The life span of nonhuman primate LAM (29 years on average for *Macaca mullata* (67)) is longer than that of rodents (36 months on average mice (68)). Nonhuman primate LAMs thus enable a more extensive longitudinal follow up in aged animals if needed, to assess long term efficacy and adverse effects of stem cell therapy.

### 3.2 Pluripotent stem cell preclinical studies in LAM

#### 3.2.1 General

The twenty-two papers included in this review span from 2011 until 2022. All use pluripotent stem cell-derived retinal cells or tissue as donor grafts for the recipient retina of a LAM eye. Data from six of the twenty-two studies (26, 69–73) indicated a two-step preclinical development process, first using rodents and then moving to a LAM model. A summary of the studies can be found in **Table 1**. One of the following two central questions were posited in all studies: (1) Do transplanted stem cells have the capacity to integrate into the host retina? (2) What immune response is elicited by transplanted cells? In addition, a focus of several studies was creating reproducible and, in two instances (26, 69), clinical-grade differentiation protocols for the stem cells used.

**Table 1:** Summary of the main features and results of LAM preclinical trials.

Aim	Donor cells	Donor cell delivery and cell dose	Features of the animal model	Stem cell delivery technique	Methods for outcome evaluation	Main findings	Reference
NHP							
To study the immunological features of iPSC-retina transplantation using MHC-homozygote monkey iPSC-retinas in monkeys with laser-induced retinal degeneration in MHC-matched and -mismatched transplantation.	mkiPSC-retinal organoids from cynomolgus monkey skin fibroblasts	Graft sheets from retinal organoids	1.NHP: cynomolgus macaques (Macaca fascicularis) RD model (577nm laser ablation) 2.Rodents: SD-Foxn1 Tg (S334ter) 3LavRrrc nude rats, C3H/HeJ mice, rd1-2J mice.	PPV, subretinal bleb formation, subretinal delivery	In vivo: color fundus photography, OCT Post-mortem: IHC	MHC-mismatched transplantation without immune suppression showed no signs of rejection and histologically showed graft maturation without lymphocytic infiltration.	74
To evaluate the therapeutic potential of photoreceptor precursors derived from clinically compliant iPSCs.	hiPSC-retinal progenitor cells generated from CD34+ cord blood	Naïve NHP: Suspension of 4x10^4^ to 6x10^4^ cells Diseases NHP: Suspension of 1x10^5^ to 3x10^5^ cells	NHP: three wild-type and three RD models (subretinal cobalt chloride injection), aged 3-5 years old and weighing 3-4.5 kg	Manually injected subretinally using an extendible 38 G injection cannula	In vivo: color fundus photography, OCT, FAF, IR Post-mortem: IHC, H&E	No adverse effects in naïve NHP models. Transplant in RD NHP models showed survive and to mature into cones post-transplant 3 months.	75
To develop a long-term, in vivo, single cell resolution monitor platform to track the behavior of transplanted PR precursor cells.	hESC-CRX^+/tdTomato^ optic vesicles	Suspension of 2.5x10^5^-1x10^6^ cells	NHP: two wild-type, three RD models (laser lesioned)	PPV, subretinal bleb formation, subretinal delivery using a 41-gauge cannula	In vivo: FAOSLO, AOSLO, OCT Post-mortem: IHC	Fluorescent reporter with FAOSLO can track transplanted PRPs in vivo. IHC showed PRPs migrated to the OPL in ablated areas.	76
To establish a preoperational evaluation system of immunosuppressive agents for the treatment of postoperative immune rejection.	hiPSC-RPE from dermal fibroblasts	Suspension of 1x10^6^ cells	NHP: wild-type	PPV, subretinal bleb formation, subretinal delivery using a 38-gauge cannula	In vivo: color fundus photography, OCT Post-mortem: IHC, H&E	With Drug-LGIR, CsA or triamcinolone successfully suppressed RPE-related immune rejections with RPE grafts without any signs of rejection.	77
Development of two medical devices for the preparation, conservation, and implantation of the hPSC-RPE sheet in NHP.	hESC-RPE	NHP: hESC-RPE sheet Rodent: suspension of 1x10^6^ cells	NHP: Wild-type Rodent: Nude mice (SOPF - BALB/cOlaHsd-Fox1^nu^) were grafted at 6-8 weeks of age.	PPV, subretinal bleb formation, retinotomy, subretinal delivery	In vivo: color fundus photography, OCT, ffERG, mfERG, slit lamp Post-mortem: IHC	Developed two medical devices for preparation, conservation, and implantation of the hPSC-RPE sheet in NHP. The surgery was safe and well tolerated during the 7-week follow up. The graft integrity was preserved in primates.	73
1. Report occurrence of acute severe inflammation after mycoplasma-infected iPSC-RPE cells implantation in NHP model 2. Determine the mechanisms of the inflammation.	iPSC-RPE generated from cynomolgus monkey skin fibroblasts	Suspension of 2.4x10^6^ cells, mycoplasma-infected	Wild-type, 2 MHC-matched and 1 MHC mismatched	PPV, posterior vitreous detachment, subretinal transplantation	In vivo: color fundus photography, FA, OCT In vitro: pyrosequencing, PCR, FACS, cytokine array Post-mortem: IHC, H&E	Mycoplasma-infected iPS-RPE cells can stimulate immune responses, thereby causing severe inflammation in the recipient eye after transplantation.	78
To assess the competency of hiPSC-retina to mature in the degenerated retinas of rat and monkey models.	hiPSC-retina derived from PBMC	hiPSC-retina pieces (0.5x2 to 1x1.5 mm^2^)	1. Rodent: SD-Foxn1 Tg(S334ter)3LavRrrc nude rats aged 2-5 months 2. NHP: aged at 8 and 6 years old, RD model (laser ablation)	PPV, subretinal bleb formation, retinotomy, subretinal transplantation	In-vivo: color fundus photography, fERG, visual field, OCT Post-mortem: IHC	Mature PR in graft survived for 2 years and a mild recovery of light perception was suggested 1.5 years after transplantation in monkey.	Tu et al., 2018
Characterization of intraocular immune response to subretinal transplantation of allogeneic miPSC-RPE in an NHP model	Allogeneic iPSC-RPE from rhesus macaque (Macaca mulatta) fibroblasts	Suspension of 5x10^5^ cells	Wild-type, female, aged 7-12 years	Transvitreal approach w/o vitrectomy, subretinal bleb formation, transscleral injection of allogenic miRPE cells	In-vivo: colour fundus photography, FAF, OCT Post-mortem: IHC	Engraftment failure of allogeneic miPSC-RPE into wild-type NHPs due to localized intraocular rejection highlights the need for autologous transplant	25
Potential of hESC retina to survive, form axons and integrate into the NHP retina following subretinal transplantation	hESC-retinal neurons	Suspension of 1x10^6^ cells	Wild-type	“Ab externo”: sclerotomy, choroidotomy, subretinal bleb creation, catheter insertion,submacular hESC-retina injection	In vivo: color fundus photography, FAF Post-mortem: ICC, IHC	hESCs-retinal neurons survived for 3 months, some integrated into the host inner retina, and formed donor axonal projections, some projecting into the optic nerve.	79
Assessment of competency and maturation timeline of hESC-retina graft in two rodent and two NHP models of retinal degeneration.	hESC-retinal neurons	Cell sheets (differentiated in a 3D culture) Cell number not specified	1. Nude rats with and without ESRD (rho mutation) 2. NHP: two ESRD models (subretinal cobalt chloride injection or photocoagulation with a 577-nm OPSL)	PPV, posterior vitreous detachment, retinal detachment with BSS, subretinal graft transplantation	In vivo: focal ERG, color fundus pictures, FA, OCT, VA evaluation with Landolt rings Post-mortem: IHC	Grafted hESC-retinal neuron sheets differentiated into a range of retinal cell types, including PRs. IHC suggested formation of host–graft synaptic connections.	70
1. Assessment of clinical-grade hiPSC-RPE cell sheets generated without artificial scaffolding and in a rat and NHP model. 2. Comparison of immunogenicity of autologous and allogeneic miPSC-RPE cell sheets in an NHP	1. hiPSC-RPE from dermal fibroblasts 2. miPSC-RPE from cynomolgus monkey (Macaca fascicularis)	Rodent hiPSC-RPE cell sheets (1.42x10^4^ cells), hiPSC-RPE suspension of 1x10^5^ cells NHP: miPSC-RPE cell sheets, 1x2mm cell sheet, cell number not specified, miPSC-RPE suspension of 5x10^4^ cells	1. RCS rat model (recessive MERTK mutation) 2. Wild-type cynomolgus monkey (Macaca fascicularis)	PPV, posterior vitreous detachment, subretinal graft transplantation	In vivo: Color fundus photography, FA, OCT	1. hiPSC-RPE cell sheets without artificial scaffolding showed characteristics similar to those of native RPE in vitro and in vivo. 2. An autologous miPSC-RPE cell sheet transplant showed no signs of graft rejection, while allogeneic miPSC-RPE cell sheets did.	69
Pig							
To test the feasibility and safety of subretinal transplantation of hiPSC-RPE into the healthy margins and within areas of degenerative retina in a pig GA model.	hiPSC-RPE generated from cord blood	Suspension of 2.5x10^5^ or 3.3x10^5^ cells	Minipigs aged 7-8 months, weighing 20-30 kg, RD model (subretinal injection of NaIO_3_ at 0.01 mg/mL or 0.1 mg/mL)	Subretinal bleb formation, retinotomy, subretinal delivery using a 41-gauge cannula	In vivo: FAF, OCT Post-mortem: IHC, H&E	Engrafted hiPSC-RPE cells formed mature epithelium in healthy retina but failed to form an epithelial-like layer in atrophic zone.	80
To assess the safety and tolerability of subretinal injection of higher than established clinical dose of hESC-derived RPE cells in minipigs	hESC-RPE	Suspension of 6x10^5^ and 1.2x10^6^ cells	Wild-type, aged 16 months and weighing 30 kg	PPV, subretinal transplantation using a 38-gauge canula	In vivo: color fundus photography,OCT Post-mortem: IHC, H&E, FISH	Subretinal injection of hESC-derived RPE cells in minipigs is well-tolerated and safe.	81
To demonstrate safety and viability of the transplant in preclinical models	hiPSC-RPE generated from PBMC of a healthy 25-year-old male	Rodent: 1 mm^2^ patch of iPSC-RPE implant Pig: 1 cm^2^ patch of iPSC-RPE implant	Wild-type	PPV, subretinal bleb formation, subretinal delivery	Post-mortem: IHC	implant allows human RPE cells to survive and maintain their phenotype and orientation without any adverse events.	71
1. GMP creation for clinical-grade, AMD patient specific iPSC-RPE patch on a biodegradable scaffold 2. Safety and efficacy testing and functional validation of iRPE patches versus cell suspension in a rodent and LAM model of ESRD.	hiPSC-RPE generated from CD34+ PBMC of three human AMD patients	Rodent: 0.5 or 1mm-diameter patch (2,5x10^3^ or 1x10^4^ cells) Suspension of 1x10^5^ cells Pig: 4x2mm patch (1x10^5^ cells) Suspension of 1x10^5^ cells	1. RCS rat model (MERTK mutation), nude rat model (strain: Crl:NIH-Foxn1rnu) 2. Pig AMD model: selective injury of pig’s visual streak RPE using 1% DC micropulse laser	PPV, posterior vitreous and retinal detachment, retinotomy, subretinal delivery of iRPE-patch loaded in the delivery tool to the transitional zone of AMD lesions	In-vivo: mfERG, OCT, fundus infrared imaging Post-mortem: IHC	Superiority of clinical grade iPSC-RPE cell patch over iPSC-RPE cell suspension in frequency, efficacy of integration as well as functionality in both rodent and porcine LAM model of retinal injury	26
To assess the feasibility and 1-month safety of RPE implantation in Yucatán minipigs.	hESC-RPE	hESC-RPE monolayer on a parylene-C membrane (6.25mm x 3.5mm)	Wild-type Yucatán minipigs	PPV, subretinal bleb formation, retinotomy, subretinal graft transplantation	In vivo: OCT Post-mortem: IHC, H&E	RPE implants were reliably placed, without implant breakage, in the subretinal space and survive as an intact monolayer for 1 month.	82
1. Evaluation of iPSC-RPE survival in the porcine subretinal space and the immune response to grafts.	Allogeneic iPSC-RPE from domestic swine (Sus scrofa domesticus) fibroblasts	Suspension of 2.5x10^5^ cells	Wild-type, in-bread, 12–14 weeks-old	PPV, posterior vitreous detachment, retinotomy, subretinal injection of iPSC-RPE into visual streak	In-vivo: N/A Post-mortem: IHC to assess for immune cells and	Survival, but no integration, of iPSC-RPE in the subretinal space of a LAM. Allogenic iPSC-RPE cells induce innate immune response.	83
Assessment of porcine iPSC and their potential to differentiate into rods in culture, and integrate in vivo	iPSC-rod PR derived from fetal pig fibroblasts (ID6 line)	Suspension of 2x10^6^ cells	Six-week-old, systemic infusion of iodoacetic acid to induce loss of rod PR	PPV, posterior vitreous detachment, subretinal bleb creation at the visual streak, iPSC-rod PR injection	In vivo: ERG Post-mortem: IHC	Subretinal injection of differentiated iPSC-PR led to integration of 1% of cells into the retina, a portion of the cells generated OS projections.	84
Rabbit							
1. Study immunogenic properties of hESC-RPE 2. Evaluate subretinal xenotransplantation of hESC-RPE on PET in rabbits	hESC-RPE	2.4x1.1 mm^2^ (implant size)	Wild-type, pigmented, aged 2-5 months and weighing 2-2.5 kg	PPV, subretinal bleb formation, retinotomy, subretinal graft transplantation	In vivo: OCT Post-mortem: IHC, H&E, TEM	Most of the hESC-RPE survived for at least 4 weeks and at least partly sustained critical RPE functions in immunocompetent rabbits.	85
To compare the efficacy of subretinally transplanted hESC-RPE cells in a wild-type rabbit model and one with GA	hESC-RPE	Suspension of 5x10^4^cells	1. Wild-type albino rabbit 2. GA rabbit model: subretinal sodium iodate or PBS injection	Transvitreal PP approach w/o vitrectomy, subretinal bleb formation, subretinal injection of hESC-RPE	In vivo: OCT, IR or multicolour-cSLO Post-mortem: IHC	Subretinal suspension transplants of hESC-RPE did not integrate into a LAM of GA, while integrating into in a wild-type LAM model	24
1. Evaluation of rhLN-matrix to support hESC-RPE differentiation. 2. Demonstrate that hESC-RPE can replace lost tissue in a LAM	hESC-RPE	Suspension of 5x10^4^ cells	Subretinal PBS injection-induced Albino rabbit model of GA	Transvitreal PP technique: subretinal injection of dissociated hESC-RPE cells No PPV	In vivo: fundus imaging, OCT and confocal SLO Post-mortem: IHC	hESC-RPE cell suspensions show long-term, functional integration as polarized subretinal monolayers that rescue overlying photoreceptor.	29
Dog							
To improve subretinal delivery and long-term survival rate of transplanted cells and promote sufficient integration into the host retina.	hESC-photoreceptor precursor cells (PRPCs) from WA09 CRX-tdTomato or WA09 NRL-EGFP cell lines	Suspension of 2x10^6^ to 4x10^6^ cells	Dogs aged 5 months to 3 years, including 7 wild-type dogs (12 eyes) and 3 rcd1/PDE6B mutant dogs (6 eyes).	Transvitreal approach w/o vitrectomy, subretinal bleb formation and delivery	In vivo: color fundus photography, cSLO, OCT Post-mortem: IHC	Transplants in systemic immunosuppressive dogs survived up to 5 months post-injection. Donor PRPCs differentiated to photoreceptors with synaptic pedicle-like structures that established contact with second-order neurons in rcd1/PDE6B mutant dogs.	27

AMD, age related macular degeneration; BSS, balanced salt solution; FA, fluorescein angiography; FAF, retinal fundus autofluorescence; GA, geographic atrophy; GMP, good manufacturing protocol; h, human; iPSC, induced pluripotent stem cells; IR-cSLO, infrared- confocal scanning laser ophthalmoscope; m, monkey; OCT, optical coherence tomography; OPSL, optically pumped semiconductor laser; PBMC, peripheral blood mononuclear cells; PLGA, polylactic-co-glycolic acid; PPV, pars plana vitrectomy; PR, photoreceptors; RCS, Royal College of Surgeons; RPE, retinal pigment epithelium.

#### 3.2.2 Target diseases

Sixteen of the twenty-two papers name a specific disease target (24, 26, 27, 29, 70–73, 75, 77, 79–83, 85). AMD was the most frequently mentioned, in Thirteen studies (24, 26, 27, 29, 71, 73, 77, 79–83, 85). Of these, five studies (71, 73, 79, 81, 83) additionally referred to Stargardt disease, macular dystrophies (Best and Stargardt disease), and retinitis pigmentosa as treatment targets, while three study (70, 72, 75), mentioned only RP as the treatment target.

#### 3.2.3 Donor cell source

Induced pluripotent stem cells (iPSCs) were utilized in twelve studies (25, 26, 69, 71, 72, 74, 75, 77, 78, 80, 83, 84), while embryonic stem cells (ESC) were used in ten (24, 27, 29, 70, 73, 76, 79, 81, 82, 85). Of the iPSC-derived cell lines, two were derived from pigs (domestic swine and pig fetus) (83, 84), four from NHP (rhesus macaque *Macaca mulatta* and cynomolgus monkey *Macaca fascicularis*) (25, 69, 74, 78) and seven from human cells (26, 69, 71, 72, 75, 77, 80). In all but one instance, fibroblasts were used as source material, but in the recent paper by Sharma et al., pluripotency was induced from human CD34+ peripheral blood mononuclear cells (PBMC), with the stated goal of limiting oncogenic mutations during cell reprogramming (26, 86). This paper also aimed to establish a potential clinical protocol for transplanting autologous iPSCs, deriving their cell lines from three AMD patients to prepare for the clinical use of autologous iPSC-derived RPE transplants and thus addressing the issue of potential immune rejection of allogeneic iPSC transplants. Kamao et al. transplanted one NHP using an autologous transplant (69). All other iPSC transplants were allogeneic or xenogeneic.

The ten groups utilizing ESC-derived retinal tissue all used human ESC (hESC) cell lines (24, 27, 29, 70, 73, 76, 79, 81, 82, 85).

Fourteen studies featured differentiation of the pluripotent stem cells into RPE cells, while the authors of the remaining eight papers (27, 70, 72, 74–76, 79, 84) aimed to replace cells of the neurosensory retina including photoreceptor cells. The predominant culturing of RPE reflects the stated aim of twelve studies to regenerate cells in non-neovascular AMD, the most common cause of blindness in the developed world (87). Eight of these studies specifically mention the goal of treating of the advanced form of dry AMD, also known as geographic atrophy (GA) (29, 71, 73, 80–83, 85). In studies aiming to replace neurosensory retina, RP is mentioned as the target disease (70, 72, 75), while Chao et al. argue that retinal neuronal replacement will be needed for GA treatment to address the secondary PR degeneration that is a typical feature in this condition (79).

#### 3.2.4 Cell formulation

Donor cell formulations were prepared in two different ways: as a suspension of dissociated cells or as a preformed monolayer. The latter approach, aiming to recapitulate normal anatomy more faithfully, was undertaken either with or without the use of extrinsic scaffolds to immobilize the cells of the monolayer. Cell suspensions were transplanted in the majority of studies.

From the nine studies featuring the transplantation of cell monolayers (26, 69–74, 82, 85), three studies transplanted both cells in suspension and as a monolayer (26, 69, 73). Six groups transplanting cell sheets included used miPSC- or hESC-RPE cells cultured without extrinsic scaffolding (69, 73), hiPSC-RPE cultured on a biodegradable polylactic-co-glycolic acid (PLGA)-based patch (26), hiPSC-RPE on nanostructured fibrin agarose (71), hESC-RPE cultured on polyethylene terephthalate (PET) membrane and parylene-C membrane (82, 85) while hESC-, hiPSC- or mkiPSC- retinal neuron sheets or cell suspension differentiated in a three-dimensional (3D) organoid culture system were used in five studies (27, 70, 72, 74, 76). Kamao et al. forwent use of an extrinsic scaffolding with the aim of avoiding potential inflammation caused by biodegradable scaffolds as well as the potential limitation of physiological interactions between choroid and RPE in nonbiodegradable membrane scaffolds (69). The mesh-supported submicron parylene-C membrane used by Koss et al. possess the advantages of ultrathin parts with diffusion, excellent RPE adherence and survival, whereas the long-term effects of its non-degradability *in vivo* is unknown (82). To examine the immune reaction to the PLGA based biodegradable scaffold, Sharma et al. first transplanted the scaffold without iPSC-RPE and did not pick up an inflammatory response on the OCT (26).

#### 3.2.5 Cell dose

The number of cells varied widely, ranging from 4 x 10^4^ – 4 x 10^6^ cells, with the average number of transplanted cells being 1 x 10^6^, and the median, 7.6 x 10^5^ cells. Four groups transplanted just about 5 x 10^4^ cells (24, 29, 69, 75), while the study with the highest number of transplanted cells used 4 x 10^6^ cells (27). All of the above-mentioned studies transplanted cells in suspension. Of the nine studies that transplanted RPE cell sheets (26, 69, 71, 73, 82, 85) or neuron sheets (70, 72, 74), four specifies the number of cells transplanted into their respective LAMs: Sharma et al. transplanted hiPSC-RPE cell sheets e1 x 10^5^ cells in their porcine NHP model (26). Koss et al. have a density of 10^5^ cells/cm^2^ in the 6.25 mm x 3.5 mm hESC-RPE sheet (82) while Garcia Delgado et al. and Ilmarinen et al. both seeded RPE at a density of 2 x 10^5^ cells/cm^2^ on their scaffolds for transplantation (71, 85).

#### 3.2.6 Recipient LAM features

The most common LAM host for iPSC- or hESC- derived donor cell transplantation was the NHP. Three different species, rhesus macaques (*Macaca mulatta*), cynomolgus monkeys (*Macaca fascicularis*) and squirrel monkey (*Saimiri sciureus*)), were used in eleven papers (25, 69, 70, 72–79). Pigs were used in seven instances (26, 71, 80–84). In the pig studies, Yucatan miniature swine, a miniature breed of the domestic pig *Sus scrofa domesticus* was used in three studies, and alternative minipig strains were utilized in two studies but in one study the strain was not specified. Albino rabbits were used in two LAM studies (24, 29), and pigmented rabbits (Chinchilla-Bastard Hybrid and Dutch-Belted rabbits) were used in one study (85). Dogs were used in one study (27).

In six studies, investigators used a two-step approach utilizing LAM only after preliminary testing in rodents. Sharma et al. tested their iPSC-derived RPE patches differentiated on biodegradable PLGA scaffolding first in RCS rat model and then in a pig model with laser-induced RPE injury (26). Kamao et al. also used the RCS rat model to determine functional and structural competency of hiPSC-RPE cell sheets prior to testing with cynomolgus monkeys (69), while Shirai et al. transplanted hESC-derived retina into nude rats with and without retinal degeneration to determine graft competency as well as the optimal differentiation day (DD) for transplantation, before introducing them into two models of retinal degeneration in rhesus macaques (*Macaca mulatta*) and cynomolgus monkeys (*Macaca fascicularis*) (70). Tu et al. reported that mature photoreceptors in hiPSC-retina graft survived well in the host retinas for at least 5 months in rat and to over 2 years in monkey. Furthermore, RGC light responses were detected at the grafted area in rat and monkey after transplantation (72). In consideration of safety, Ben M’Barek et al. performed the transplantation prior to NHP and found that the hESC-RPE sheet neither induced teratoma nor dispersed to other organs in nude rodents (73) In another study, the lack of damage of the host retina in addition to survival of transplanted cells in mice formed the basis to pursue transplantation of iPSC-derived RPE in pigs (71).

A significant challenge in working with LAMs as a translational model is the lack of naturally occurring models for retinal disease. As a result, almost half of the studies utilized wild-type models: Sohn et al. used the Yucatan miniature swine model to evaluate iPSC-derived RPE from domestic swine fibroblasts (83). Koss et al., Garcia Delgado et al., and Cho et al. assessed the safety of the RPE transplant in wild-type pigs (71, 81, 82). Chao et al. transplanted hESC-derived retinal neurons into a wild-type squirrel monkey (79), McGIll et al. transplanted allogeneic iPSC-derived RPE into non-immunosuppressed rhesus monkeys (25). Kamao et al. used wild-type cynomolgus monkeys as a recipient model for miPSC-derived RPE sheets and cell suspension (69). Makabe et al., Ben M’Barek et al., and Fujii et al. also had wild-type NHP for transplantation (73, 77, 78). A wild-type rabbit model was used by Petrus Reurer et al. in conjunction with the rabbit model of GA (24) while Ilmarinen et al. only used wild-type rabbits in transplantation (85).

In the studies that utilized disease models, damage was induced exogenously to mimic human disease features. To this end, retinal injury was induced systemically or locally by chemical or photic means. Systemic administration of a retinotoxic pharmacologic agent was used in only one study by Zhou et al., where iodoacetic acid was administered to injure RPE and PR cells of pigs (84). Local subretinal injection was a more commonly used approach to model features of retinal degeneration: sodium iodate (NaIO3) or phosphate-buffered saline (PBS) were used to injure PR and RPE (24, 80), and cobalt chloride was used to selectively injure PR (24, 70, 75).

Laser-induced injury was used in four models: a 1% duty cycle (DC) micropulse laser as well as a 577-nm optically pumped semiconductor laser (OPSL) which induced thermal photocoagulation of RPE and photoreceptor cells while preserving INL (26, 70, 72, 74). A 730-nm ultrafast laser emitted short pulses of light (55fs) and a continuous wave (647-nm) laser were also used to create localized lesions in the photoreceptor layer (76). Ripolles-Garcia et al. used the sole genetic mutation model included in this review: a dog model with rod-cone degeneration caused by a nonsense mutation in the *PDE6B* gene (rcd1/*PDE6B*) (27).

#### 3.2.7 Surgical technique

In the studies included in this review, all pluripotent stem cell derivatives were delivered subretinally, with the goal of ultimately replacing PR or the RPE. The investigators vitrectomized the LAMs eye in fifteen studies (26, 69–75, 77, 78, 81–85), while this procedure was not performed in six reported studies (24, 25, 27, 29, 79, 80). Aboualizadeh et al. transplanted cells in three monkeys without vitrectomy and another two monkeys with vitrectomy (76). It is notable that although Ripolles-Garcia et al. injected cells without performing vitrectomy, they recommended a 5-step subretinal injection technique (including vitrectomy) to reduce vitreal reflux after injecting cells in the subretinal space (27).

In the thirteen studies using cell suspensions, some investigators opted to form a subretinal bleb with BSS (24, 70, 75, 77, 84) or, in one case, Healon (79) before infusing the stem cells, while others immediately infused the stem cells without pre-implantation bleb induction (27, 29, 76, 78, 80, 81, 83). One study, that of Chao et al., used an “ab externo” technique, rather than a transvitreal technique, which required a sclerotomy and a choroidotomy prior to subretinal bleb creation with Healon and subsequent stem cell derivative infusion (70). In the nine studies using cell sheet monolayers with or without scaffolds, the sheets were introduced subretinally via the transvitreal approach (26, 69–74, 82, 85).

#### 3.2.8 Outcome evaluation assays

All investigators used post-mortem immunohistochemistry (IHC) of enucleated eyes to evaluate graft integration and/or immune responses postmortem. *In vivo*, multifocal electroretinography (mfERG) was used as an objective functional outcome measure. In Shirai et al., NHPs were trained to distinguish Landolt circles from complete rings as a subjective functional outcome measure to assess approximate visual acuity (VA) (70). Fundus photography, fundus autofluorescence (FAF), fundus angiography, optical coherence tomography (OCT) and scanning laser ophthalmoscopy (SLO) were also used as structural outcome measures.

#### 3.2.9 Outcomes of LAMs studies

Of the fourteen papers that transplanted RPE cells (24–26, 29, 69, 71, 73, 77, 78, 80–83, 85), most transplanted cell suspensions, but two used the RCS rat model of IRD (due to a recessive *MERTK* mutation) as well as a nude, immunocompromised rat (strain: Crl: NIH-*Foxn1* ^rnu^) to compare transplantation of cell suspensions with transplantation of cells in monolayer prior to transplantation into the respective LAM (26, 69).

Sharma et al. showed that cells in monolayer integrated more frequently and more efficiently than their cell suspension counterparts in two preclinical rodent models. They also demonstrated that hiPSC-RPE cells in monolayer showed no signs of tumor formation, whereas their cell suspension counterparts formed tumors in 1/3 of transplanted cases. When moving into a porcine LAM, the human clinical dose of a hiPSC-RPE patch was able to be used to further prove that transplantation of the monolayer hiPSC-RPE patch was superior to cell suspension in a LAM (26).

Kamao et al. used cell sheets generated without artificial scaffolding on a temporary type I collagen gel which was subsequently degraded by a collagenase in order to avoid the inflammation that might be generated from biodegradable scaffolding and the separation of the RPE from the choroid that would be the consequence of non-degradable scaffolding. In a head-to-head comparison between transplantation of cell suspension and cell sheets into cynomolgus monkey (*Macaca fascicularis*), Kamao et al. observed reflux of transplanted cells in suspension into the vitreous cavity and accumulation of transplanted cells at the lower margin of the induced retinal detachment. In contrast, cell sheets remained situated at the transplant site of transplantation, did not disperse into the vitreous cavity and could be observed without the addition of a fluorescent protein to the cells, which had been necessary to trace the cells in suspension (69).

Petrus-Reurer et al. compared the ability of a cell suspension transplant to integrate into a wild-type rabbit model versus its ability to integrate into a rabbit model with GA-like atrophy. While the cell suspensions formed monolayers and integrated into the wild-type retina, they failed to integrate into the diseased retina, leading to the hypothesis that a well-conserved ONL/RPE complex is essential for effective graft integration (24). Another rabbit study from Ilmarinen et al. found that the hESC-RPE survived for at least 4 weeks after transplantation, and at least partly sustained critical RPE functions in wild-type rabbits (85).

In keeping with those results, Sohn et al. was the first to show a survival of the transplanted iPSC-RPE suspension in the subretinal space of a wild-type porcine model 3 weeks post injection, but while the grafts survived they appeared to fail to integrate into the host RPE layer (83). In one study by McGill et al., the integration of allogeneic iPSC-RPE cell suspension into a non-immunosuppressed NHP failed, most likely due to an immune response (25). Contrary to these observations, Plaza-Reyes et al. demonstrated that hESC-RPE cell suspension showed long-term, functional integration as polarized subretinal RPE sheet that rescued overlying PR function in the rabbit GA model (29).

Sharma et al. used a biodegradable PLGA scaffold. They observed not only successful integration of up to 70% of transplanted cells in both the porcine model with laser-induced RPE ablation in the visual streak as well as into the RCS rat model, but also the absence of teratoma formation in the RCS rat model. This low oncogenic potential was mirrored in the absence of the proliferation marker Ki67 on IHC. Ten weeks post-transplant, it was confirmed that 70% of transplanted cells survived and that the PLGA scaffolding had degraded successfully. Also, the integrated patch was successful in maintaining both the inner and the outer retina as reflected in the recovery of mfERG signals over the transplanted patch (26).

Koss et al. utilized a mesh-supported submicron parylene-C membrane to culture the hESC-RPE monolayer and proved the feasibility and safety of their transplants in wild-type pigs (82). Ben M’Barek et al. embedded RPE sheets into gelatin to keep the RPE monolayer structure in transplantation (73). Garcia Delgado et al. selected a nanostructured fibrin-agarose hydrogel (FAH) to support RPE and successfully transplanted iPSC-RPE-FAH patch in pigs without any adverse events (71). One advantage of having scaffold-supported RPE monolayer is that the polarized RPE with the proper orientation is critical to maintain the health and integrity of photoreceptors. Conversely, Duarri et al. found that the hiPSC-RPE cell suspension failed to form an epithelial-like layer at atrophic zone in pig (80). Despite of the structure, Cho et al. transplanted single RPE cell suspension and demonstrated the subretinal injection of the cells are safe and well-tolerated (81).

Seven groups transplanted neural retinal cells (70, 72, 74–76, 79, 84). Of these, Lingam et al., Aboualizadeh et al., Chao et al. and Zhou et al., used cell suspensions, while Uyama et al., Tu et al., and Shirai et al. used cell sheets. In the two instances of transplanting retinal neurons in suspension (79, 84), both reported successful transplant integration, with Zhou et al. reporting that 1% of neurons in cell suspension integrated into a porcine model of rod PR degeneration (84).

Chao et al. observed an appearance of donor cell integration into the inner retina of one wild-type NHP with numerous donor axonal projections throughout the recipient site. Shirai et al. transplanted hESC- retinal neuron cell sheets, into four eyes of three NHP with induced retinal degeneration. They observed the grafted retina differentiating into a range of retinal cell types, as well as the formation of putative host-graft synapses, but noted the absence of axonal projections (79). Interestingly, Lingam et al. also observed the transplanted photoreceptor precursors have differentiated into cone photoreceptors in CoCl_2_-induced retinal degeneration NHP models which implies that the subretinal space provide a niche for photoreceptor precursors differentiation even in retinal degeneration models. They used a Current Good Manufacturing Practice grade (cGMP) iPSC line in their study (75). What’s more, Aboualizadeh et al. found that the hESC-derived donor photoreceptors structurally made synaptic connections with host NHP bipolar cells (76). Ripolles-Garcia et al. also observed donor PRPCs differentiated to photoreceptors with synaptic pedicle-like structures that established contact with second-order neurons in rcd1/*PDE6B* mutant dogs (27). Tu et al. further have collected the host retinal light responsiveness in NHPs, although conclusive discrimination of graft originated responses from remaining host cell activities was difficult to obtain with these models (72).

#### 3.2.10 Immune responses

Three groups (Sohn et al., McGill et al., and Kamao et al.) specifically reported their data related to the immune response to autologous and allogeneic stem cell transplants (25, 69, 83).

McGill et al. characterized the immune response to allogeneic iPSC-RPE cells and subsequent graft failure in the most detail, describing a T-cell dominant response at three days, which was converted to a B-cell dominant response at four weeks and a subsequent complete apparent loss of donor cells (25).

Kamao et al. did the only autologous transplant of the nine studies, when transplanting NHP iPSC-RPE cells back into the same donor NHP (69). Here, they were able to show an absence of rejection of the autologous cell graft. Consistent with the findings of McGill et al., indicators of early T cell activation were found in the vitreous of transplanted NHP eyes (25). Kamao et al. showed that INF-gamma, an inflammatory cytokine presumed to be elicited by transplant surgery can increase major histocompatibility complex (MHC) and beta-2 microglobulin expression on hiPSC-RPE cells, thus increasing the chances of allograft rejection (69). When comparing the PBMN cell response to an allogeneic versus an autogenic miPSC-RPE transplant in culture, Kamao et al. showed that while the MHC mismatch in between the allogeneic transplant and the PBMN cells produced an immune response, whereas the autogenic transplant did not (69). Also, when comparing an autograft with an allograft transplant, the authors found signs of rejection in the autografts (manifesting as fibrous tissue formation, fluorescein leakage, and retinal edema), which were absent in the allografts.

Sohn et al. also described an innate immune response to subretinally injected iPSC-RPE cells, which suggested a relative intolerance of the host to allogeneic donor transplanted cells (83). All three studies came to conclusion that due to their supposed virtually identical match with the recipient, autologous cell transplants might prove to have the highest chance of eliminating graft failure due to immune rejection.

Regarding retinal neuronal transplant, Uyama et al. showed that transplanted MHC-matched mkiPSC-retinal organoid sheets survived with no detectable clinical signs of rejection or lymphocyte immune reaction in NHPs. However, subclinical rejection was found in MHC-mismatched transplantation studies, which may ultimately affect long-term survival and functional integration (74).

It is possible that the stem cell-derived donor cells be contaminated by *mycoplasma* infection during the *in vitro* culturing before transplantation. Makabe et al. reported that the *mycoplasma* contaminated mkiPSC-RPE cells caused severe inflammation and immune response after transplantation in NHPs. The initial signs included retinal vasculitis and subretinal fluorescence leakage on angiography. Infiltration of inflammatory cells including Ly6G^+^ cells and NKG2A^+^ cells in the grafted area may reflect the pathophysiolic changes related to *mycoplasma* infection. This study provides additional data to suspect cases of *mycoplasma* infection in transplantation experiments (78).

#### 3.2.11 Protocol development

In order to translate preclinical studies into the clinic, the establishment of good manufacturing practices (GMP) for stem cell therapy is a key factor. Establishing a standardized protocol for this regenerative therapy is a critical factor in providing consistent and safe treatment with reproducible results. Hence several of the studies focused on creating and streamlining a manufacturing protocol that would move toward such clinical-grade requirements.

Sharma et al. established a clinical-grade, safe and efficacious iPSC differentiation protocol that used CD34+ PBMN to create autologous iPSC-RPE cell lines from three AMD patients. Cells were differentiated on PLGA based biodegradable scaffolds, making it possible for them to be transplanted in an immobilized monolayer (26). The preclinical study by Sharma et al. is in the process of completing an investigational new drug application for a phase I trial (88).

As described above, Kamao et al. set up a protocol for developing clinical-grade hiPSC-RPE cell sheets that did not feature an artificial scaffold (69). The autologous hiPSC-RPE sheets then were used for treating a neovascular AMD patient, and no serious adverse event was observed at 25 months post-transplantation (2). Further study implanted HLA-matched allogeneic iPSC-RPE into five wet AMD patients showed stable survival and safety of iPSC-RPE transplantation for a year (89). A similar methodology was used by Shirai et al. in 2016 when the clinical utility of hESC-retina was assessed using neural retinal sheets derived from a 3D organoid culture system (70).

Plaza Reyes et al. set out to establish a xeno-free hESC-RPE differentiation protocol and successfully used a recombinant human laminin (rhLN) and E-cadherin matrix to support the differentiation of hESC into RPE (29). Zhou et al. devised a two-step differentiation protocol for photoreceptor generation from iPSCs (84).

Koss et al. demonstrated the feasibility of the surgical techniques to place the hESC-RPE monolayer with the mesh-supported submicron parylene membrane into the subretinal space of the pigs. Furthermore, the hESC-RPE cells were found to be survived as an intact monolayer at 1-month after transplantation (82). Thereafter, the developed hESC-RPE transplants were implanted to four dry AMD patients in a phase 1/2a study (4). Remarkably, none of the implanted eyes showed progression of vision loss by the metrics studied, while one eye improved by 17 letters and two eyes demonstrated improved fixation one year after surgery.

### 3.3 Discussion

#### 3.3.1 Relevance of LAM ocular morphology to surgical modeling

Due to the gross anatomical discrepancies between rodent and human eyes, surgical technique and instrumentation established in rodents have only limited use for direct translation into human trials. The emerging conceptual trend is for stem cells to be transplanted as preformed monolayers rather than as dissociated cell suspensions. The surgical technique of the former approach is somewhat more complex than for the latter – requiring larger incisions and more delicate tissue handling, transfer, placement and immobilization maneuvers. Hence, the establishment, evaluation, and iterative optimization of the surgical protocol has become integral to the success of any clinical stem cell trial. In fact, the majority of adverse events that have been reported in retinal stem cell therapy clinical trials can be generally referred to the surgical delivery rather than the biologic being investigated. Due to their similar anatomical size to human eyes (see **Figure 1**), LAMs allow more direct translation of surgical instrumentation and techniques into human clinical trials, therefore being valuable tools for the successful translation of preclinical studies to the clinic.

#### 3.3.2 Age-related macular degeneration as a disease target

AMD is the most frequently targeted disease in these preclinical LAM studies, reflecting the fact that AMD is the most frequently targeted disease in stem cell trials to date (11). The results of several landmark Phase I/II clinical trials, including the first-in-human pluripotent stem cell transplant for advanced dry AMD (1, 16, 17) as well an autologous iPSC transplant in a patient with acute wet AMD (2), have been reported. Furthermore, generally, transplanting RPE is fraught with fewer scientific hurdles than replacing retinal neurons: there is no need for formation of neuronal connections and integration into neuronal networks in the case of RPE transplants. But the treatment of GA presents the challenge of late-stage PR degeneration in areas of atrophy, so that not only the RPE but also the overlying PR might have to replaced (90) for vision to be restored effectively.

#### 3.3.3 Importance of pluripotent stem cell technology in retinal therapy development

As primary cells isolated from the fetal retina present certain disadvantages, including ethical concerns and limitations in the availability of material, recent years have seen the development of protocols to obtain transplantable photoreceptors and RPE from hESCs and iPSCs by using 2D and 3D differentiation culture systems (91). With the advent of iPSC cells in 2007, the ability to create patient–specific autologous transplants was developed. While anterior chamber associated immune deviation (ACAID) was long thought to applicable to the subretinal space, this immune privilege is being re-examined in the context of retinal therapy applications (92–94), in particular after subretinal manipulation. Hence, the idea of a genetically identical, autologous transplant that would not induce an immune response, at least in theory, is very attractive. Although only one autologous transplant was conducted among the nine studies, all studies describing an immune response in allogeneic transplants concluded that autologous cell transplants may improve the success of transplanted grafts. Although autologous transplants carry hope to decrease immune response, it might be posited that these pose a risk for AMD recurrence, since none of the predisposing genetic or environmental factors were targeted. However, a mitigating factor in this regard is that the age of the transplanted cells could be regarded conceptually as having been reset to a more juvenile stage prior to transplantation, when cellular features of AMD do not typically develop *in situ*. It is surprising that hESC were used almost as frequently as iPSC in these preclinical studies, considering that iPSC cells are thought to raise less significant ethical concerns, are more readily available, and allow for a non-immunogenic autologous patient transplant. One possible reason could be the concern regarding the variability and genetic instability of iPSC lines, whereas ESCs are considered generally more consistent in their genetic and epigenetic profiles and represent the ‘gold standard’ in pluripotent stem cell research (95). The utilization of engineered pluripotent stem cells (e.g., HLA-E gene knock in) to mitigate immune rejection hold potential for future application though these have not yet been studied in the LAM models (96). In light of concerns about inefficient integration of cell suspensions (16) and their high immunogenicity (97), the number of studies using cell suspensions rather than cells in monolayer was also surprisingly high. This might simply stem from the increased technical difficulties of administering cells as monolayers, which include the need to develop a suitable scaffold as well as complex surgical transplantation technique. The properties of Bruch membrane serve as a basis for scaffold material selection for tissue-engineered RPE cell monolayers (98). A wide variety of scaffolding materials in these studies have validated the safety and tolerability of various combinations of cells and materials.

#### 3.3.4 Porcine versus nonhuman primate models

The porcine eye is the closest in size to the human eye among large animal models (LAMs), making it an ideal model for testing surgical methods of donor graft transplantation. Additionally, the expense is less than that of non-human primates (NHPs), and pigs are readily available (presumably due to their widespread use in agriculture), helping to ensure a relatively steady supply. Due to its approximate ocular dimensions being close to the human eye, yet its relatively small overall physical size, the rabbit model combines the positive features of rodent models, such as low maintenance costs and ease of breeding, while offering a more realistically scaled model of the human eye. These characteristics also could facilitate using higher sample sizes to effectively address the research questions with adequate statistical power. In terms of physiological modeling of the human eye, NHPs most closely replicate the distribution of rods and cones in the human macula, making them best suited as models for diseases affecting the foveomacular region, particularly when the research focuses on foveal cell characteristics. However, some aspects of the use of NHPs in ophthalmology research can be controversial due to ethical concerns regarding their cognitive abilities and the impact of captivity on their well-being. Furthermore, research involving NHPs is subject to stringent regulatory oversight, which can potentially slow down the research process and thus increase costs. Additionally, NHP research typically entail higher costs than other LAMs due to the need for specialized group housing, veterinary care, and environmental enrichment strategies.

#### 3.3.5 Canine and feline models

Dogs, which have been a popular LAM choice in retinal gene therapy preclinical studies (66, 99, 100), have been rarely used in LAM pluripotent retinal stem cell studies. The crucial advantage dogs show over other LAM in preclinical gene therapy trials is that they harbor several monogenetic retinal mutations, in *BEST1, RPGR* or *RPE65* to name a few. These variants often cause a disease progression and pattern that mirrors aspects of the human disease course (66). Recently, Ripolles-Garcia et al. have reported the transplantation of hESC-derived photoreceptor precursor cells in both wild-type dogs and rcd1/*PDE6B* mutant dogs (71). They demonstrated that systemic immunosuppression using oral cyclosporine A (CsA), and mycophenolate mofetil (MMF) preserved long term survival of transplanted photoreceptor precursors. Donor cells remained mostly in the subretinal space in the wild-type dogs, while they had extended axons in outer limiting membrane (OLM)-disrupted mutant dogs, suggesting that the disruption of OLM may enhance the donor cell integration. Since stem cell therapy is focused on replacing degenerated retina regardless of cause, the importance of these mutations-specific models could be considered secondary to the value of the anatomical and physiological similarities of LAM to human eyes. Similar to dogs, cats also have a holangiotic retina and lack a macula, instead possess a visual streak and a region analogous to the macula known as the area centralis. This area, rich in cone photoreceptors, is essential for high-acuity vision and has higher metabolic demands (101, 102). The limited use of dogs and cats in the transplantation field may be attributed to several factors: the size of the pig’s eyeball more closely resembles the human eye compared to that of dogs and cats, the absence of a true macula or fovea in dogs and cats, and public sentiment regarding the use of these domestic companion animals for invasive surgery research (40, 103).

#### 3.3.6 Stringent regulation of nonhuman primate research

In the USA and other jurisdictions, research involving the use of non-human primates is regulated at several levels, including federal, state/local, and institutional. In particular, the USDA regulates the use of NHP and other large animal species for research, and issues breeders’ and dealers’ licenses to research centers. Funding sources can impact the regulatory bodies that govern certain types of research. The USDA also inspects research facilities regularly to ensure compliance with standards in addition to requiring them to submit annual reports to maintain licensure. In addition to the approval and licensure of the research facility, individual investigators and researchers must meet the training requirements set by their Institutional Animal Care and Use Committee (IACUC). The IACUC reviews and approves experimental procedures and protocols involving the use of animals in research. In cases where principal investigators are not experienced in research involving NHP, the IACUC requires them to collaborate with other experienced investigators. It also ensures the availability of trained veterinary staff to provide general support as well as advice on surgical techniques to be used. Research staff also needs to obtain clearance from occupational health including compliance with testing and vaccine requirements.

#### 3.3.7 Evolving role and use of LAMs in retinal therapy development

The role of LAMs in advanced therapy medicinal product (ATMP) development will almost certainly continue to evolve as testing in large animal models (LAMs) is an crucial step in translating retinal ATMPs into patients. Ideally, LAM experimental design should be driven by scientific goals. However, challenges including budgetary and infrastructure constraints can hinder LAM research and modify its scope. The anatomical similarity of LAM and human eyes allows the implementation of clinically-relevant surgical techniques as a preamble to their application in human study participants. While the FDA Modernization Act 2.0 has provided a framework to consider alternative methods chiefly for pharmacologic studies, including tissue-on-a-chip and human cell culture models, LAM testing is generally considered useful for cell and tissue replacement studies.

### 3.4 Conclusion

LAMs are an invaluable tool for research and development on the translation of retinal stem cell therapies from the laboratory to the clinic. The most common LAMs used for retinal cell therapy research are NHP and pigs, whereas rabbits and dogs have been used less frequently. In these models, varying cell doses have been tested, ranging from 40,000 to 4,000,000 cells per administration. Due to physiological and structural similarities to aspects of the human eye, the eyes of LAMs are useful to answer preclinical research questions regarding the most efficient donor cell configuration, assessment of donor graft integration into host retina, as well as to evaluate aspects of the host immune response to the donor grafts. In addition, in general, due to their anatomical and morphological similarities to the human eye, LAMs are useful to enable the development of surgical approaches and techniques for administering ATMPs. In particular, the size of the pig eye is a useful analogue of human anatomy, while the NHP uniquely provides a macula that is lacking in other species. While alternative platforms (such as human cell culture or tissue-on-a-chip) can be used for drug and biosimilar testing, the use of animal models (LAMs in particular) play an important role in meeting the research and development goals of ATMP testing including cell and tissue regeneration modalities.

## Data Availability

The original contributions presented in the study are included in the article/supplementary material. Further inquiries can be directed to the corresponding author.
